# Exploiting Methyl Triazenes as Attractive Alternatives to Temozolomide and Dacarbazine for Cancer Therapy

**DOI:** 10.3390/molecules31071103

**Published:** 2026-03-27

**Authors:** Goreti Ribeiro Morais, Gabriel C. Nwokolo, Harriet N. L. Lamptey Mills, Richard T. Wheelhouse, Robert A. Falconer

**Affiliations:** Institute of Cancer Therapeutics, University of Bradford, Bradford BD7 1DP, UK; g.c.nwokolo@bradford.ac.uk (G.C.N.); r.a.falconer1@bradford.ac.uk (R.A.F.)

**Keywords:** prodrugs, aryl triazenes, synthetic strategies, metabolism, decomposition, hybrids, targeting, anticancer activity

## Abstract

Temozolomide and dacarbazine are untargeted anticancer prodrugs that have been widely employed in the treatment of melanoma and glioblastoma. These agents decompose into a short-lived monomethyl triazene intermediate, culminating in the release of a methyl diazonium cation that serves as the DNA-alkylating species responsible for tumour destruction. However, due to their high chemical lability, these agents have been associated with chemotherapy resistance, mutagenicity, tumour relapse, and significant off-target toxicity. One promising strategy towards the resolution of these limitations involves the design of arylmethyl triazene prodrugs, which enable targeted tumour-specific drug delivery. This review explores the various approaches used to selectively deliver alkyl aryl triazenes as alternatives to current therapies. It highlights early chemical strategies such as *N*-acylation and etherification of monomethyl triazenes, along with associated kinetic studies. The selective activation of novel triazenes in murine and human melanoma cells through a tyrosinase-responsive promoiety is discussed. Recent progress in nitroaromatic-based prodrugs designed to exploit the hypoxic microenvironment of glioblastoma is also examined. Additionally, we summarise the development of combi-triazenes and their underlying chemistries, which enable the simultaneous release of two active therapeutic agents.

## 1. Introduction

**Scheme 1:**
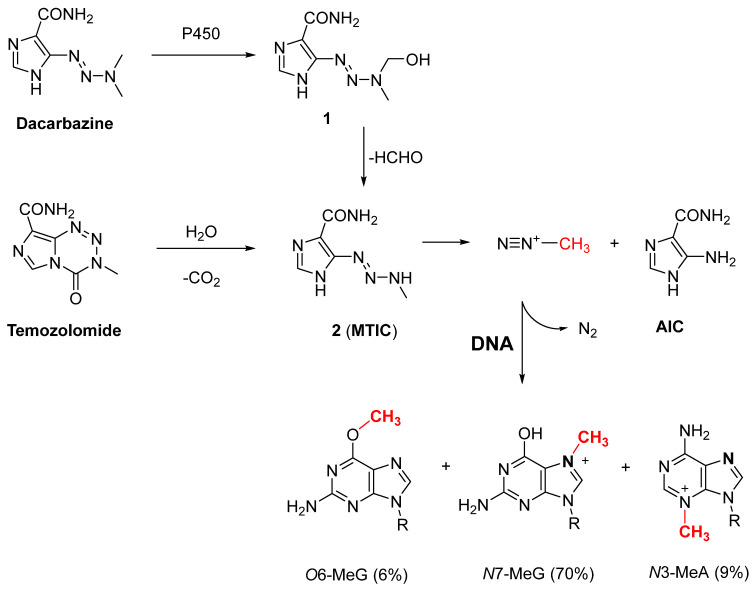
Activation and mechanism of action of dacarbazine and temozolomide [3].

**Figure 1:**
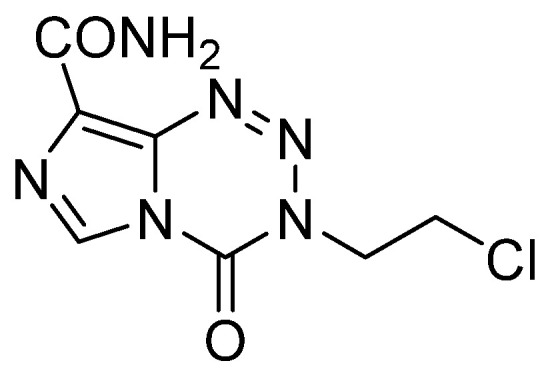
Chemical structure of mitozolomide (MTZ).

Methyl triazenes such as dacarbazine (DTIC) are an important class of compounds with significant clinical interest in cancer. Oxidation of DTIC by the liver cytochrome P450 (CYP) enzymes yields *N*-demethylated 5-(3-methyl triazen-1-yl)imidazole-4-carboxamide (MTIC) [1]. In contrast, temozolomide (TMZ) is an imidazotetrazine alkylating agent which is spontaneously hydrolysed into the same MTIC under physiological pH without the need for activation in the liver [2]. MTIC subsequently fragments (*t*_1/2_~2 min) into the inactive analogues aminoimidazole carboxamide (AIC) and methyl diazonium, with the latter spontaneously releasing molecular nitrogen and a methyl carbocation, a highly electrophilic species that alkylates the DNA to produce different ratios of guanine- and adenine-methylated adducts, resulting in cell death (Scheme 1). Despite the differences in their activation, both DTIC and TMZ generate the same methylation agent [3], and both are used in the management of advanced melanoma, soft tissue sarcoma [4], and glioblastoma (GBM) [5]. In addition to DTIC and TMZ, mitozolomide (MTZ) (Figure 1), the 3-chloroethyl analogue of TMZ, has been reported, but despite an initially exciting preclinical profile, its clinical progress was hampered due to significant toxic side effects [6].

**Scheme 2:**
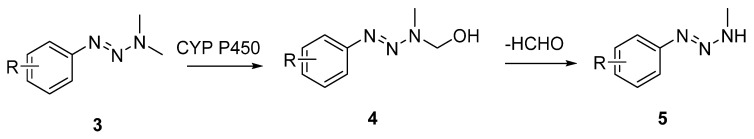
Oxidative metabolism of aryl dimethyl triazenes **3** under physiological conditions [12].

The main difficulty with non-selective prodrugs such as DTIC and TMZ arises from their non-discriminatory cytotoxicity, which extends beyond tumour cells to destroy normal cells, resulting in significant side effects that undermine their clinical utility [7]. Furthermore, DTIC not only possesses modest pharmacological effectiveness as a single agent, partly due to the lower activity of CYP enzymes in humans compared to rodents, but also displays chemical instability and susceptibility to photodecomposition [8], as well as a characteristic formation of toxic diazonium salts, which render it a substantial therapeutic liability [9,10]. Comparatively, TMZ, a small (194 Da) and highly lipophilic molecule that is rapidly absorbed after oral administration [2], is also characterised by reduced pharmacological efficacy as a result of its short half-life (1.8 h), as it is almost entirely cleared from plasma within 8 h of administration [11]. A strategy to enhance the bioavailability, improve efficacy, and minimise systemic toxicity of DTIC and TMZ involves the design of specific prodrugs related to compound **3** (Scheme 2) capable of releasing the monomethyl triazene **5** of the MTIC type specifically at the tumour site. To identify prodrugs that not only by-pass oxidative metabolism as the activation mechanism of the aryl dimethyltriazenes **3** (Scheme 2) but also achieve a controlled release of monomethyltriazene, various derivatives of **4** and **5** have been synthesised and studied over recent decades.

**Scheme 3:**
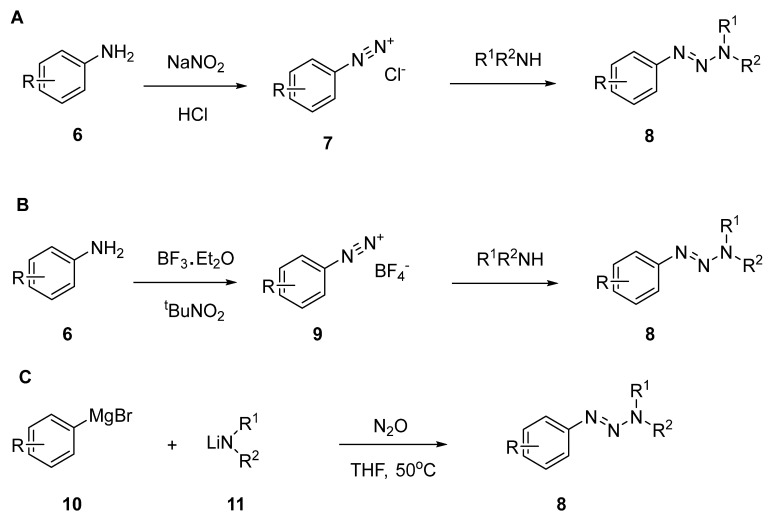
General synthetic methods to obtain aryl triazenes. (**A**) via diazonium chloride with amines; (**B**) via diazonium tetrafluoroborate with amines; (**C**) via nitrous oxide-mediated coupling of dialkyl lithium amines and Grignard reagents.

Common synthetic routes to obtain aryl triazenes involve the reaction of anilines **6** with a nitrite ion under acidic conditions to form the diazonium salt **7**, which is then quenched with a primary or secondary amine (Scheme 3A) [12]. This method requires a strong electron-withdrawing group in the phenyl ring (R = *o*-,*m*-, and *p*-NO_2_; *o*-, *m*, and *p*-CO_2_R^1^; *p*-CN and *p*-COCH_3_) to prevent the formation of pentaazadiene or 1,3-diaryltriazene by-products instead of the desired aryl metyltriazene **8** [13]. However, the challenges associated with this method are the low stability of the diazonium chloride salts, which may impose a constraint on compound storage, and by extension, their large-scale application [14]. Alternatively, the aryl diazonium can be prepared as a tetrafluoroborate salt **9**, accomplished by reacting anilines with *tert*-butyl nitrite in the presence of boron trifluoride etherate (Scheme 3B) [15]. An alternative innovative route to 1-aryl-3,3-dialkyltriazenes, in good to excellent yields (71–94%), involved the nitrous oxide-mediated coupling of dialkyl lithium amines and aryl magnesium compounds (Scheme 3C). This new method established that aryl Grignard reagents can be used as C nucleophiles, predicating the extension of their use to alkynyl and alkenyl analogues [16]. Unfortunately, the use of gaseous nitrous oxide as the nitrogen donor in this step poses a significant challenge, as the reagent is not routinely available. Subsequently, aryl magnesium halides were reacted with organic azides, but this was only demonstrated for the synthesis of 1-aryl-3-cyclic triazenes [17]. More recently, a nonradical base-promoted substitution of arylazo sulfones and amines generated disubstituted and trisubstituted triazenes, furnishing a stable alternative approach to classic triazene synthesis [18]. Moreover, arylazo sulfones can be prepared from anilines and possess the desirable advantage of higher stability to storage under ambient conditions than the aryldiazonium salts [19].

**Scheme 4:**
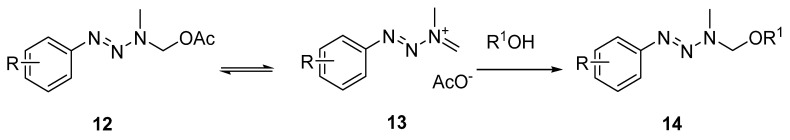
Synthesis of 3-alkyloxy-3-methyltriazene via an iminium salt [26].

The development of new 1-aryl-3-methyltriazene prodrugs usually involves the incorporation of **4** and **5** (Scheme 2) as raw materials, as exemplified by the Rosa [20,21,22,23] and Vaughan groups [24,25,26], who designed ether and thioether derivatives of the short-lived hydroxymethyl triazene **4** as prodrugs for the active metabolite monomethyltriazene **5**. Unlike the hydroxymethyltriazenes **4**, ether and thioether derivatives demonstrate better stability in aqueous media at physiological pH, highlighting their potential as viable prodrug candidates. Methoxymethyltriazene displayed antitumour activity in vivo against certain tumours while lacking activity in vitro, suggesting the presence of a biological mechanism necessary for the activation of the methoxymethyl group to the active metabolite [24]. The synthesis of those derivatives was straightforward and was achieved by the nucleophilic attack of the alcohol on an iminium species, which is in equilibrium with the 3-acetoxymethyl analogue **12** (Scheme 4) [26]. One impediment to this group of prodrugs derives from the observation that despite maintaining chemical stability under neutral and basic conditions, the ether prodrugs were found to be susceptible to decomposition under slightly acidic media, a factor that effectively hinders their clinical applicability [22].

**Scheme 5:**
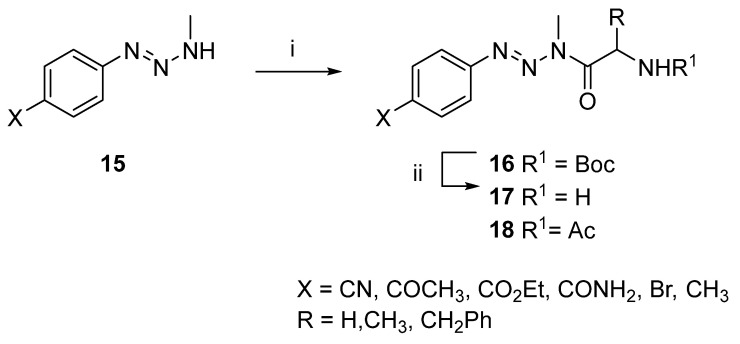
Synthesis of aminoacyl triazenes **17** and **18**. Reagents and conditions: (i) *N*-Boc amino acids, dicyclohexylcarbodiimide (DCC), dimethylaminopyridine (DMAP); (ii) HCl, CH_3_NO_2_.

**Figure 2:**
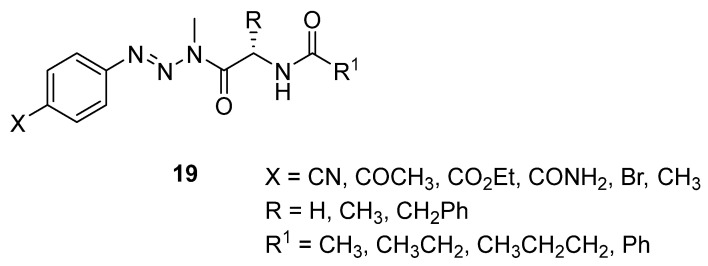
Chemical representation of *N*-(acylamino)acyl triazenes **19** [29].

Like the ether analogues **14**, the prodrugs of **5** functionalized with a 3-acyl group, by a conventional dicyclohexylcarbodiimide (DCC) coupling reaction (Scheme 5), readily decomposed into the respective anilines under acidic or basic conditions, rendering them unsuitable as prodrugs [27,28]. On the other hand, acylation of the α-amino group, as in prodrugs **18** and **19** (Figure 2)**,** was a good strategy to reduce the chemical reactivity of these triazene derivatives while retaining the rapid rate of enzymatic hydrolysis [29,30]. Compared to TMZ (*t*_1/2_ = approx. 2 h in both pH 7.4 phosphate buffer), triazenes **19** showed higher chemical stability (*t*_1/2_ = approx. 8–84 h). Modulation of log P was also accomplished using α-(acylamino)acyl derivatives **19**, ensuring effective absorption through biological membranes [29]. The kinetic parameters of triazenes **19** suggested that they are promising candidates for prodrug development; however, the lack of a targeting trigger limited further investigations.

**Scheme 6:**
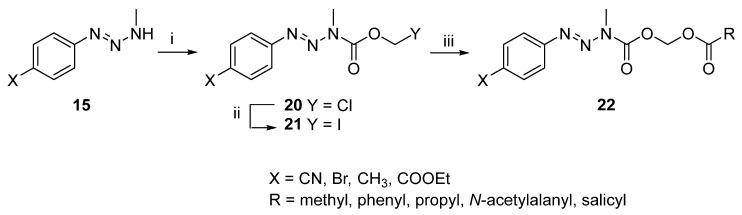
Synthesis of 3-(acyloxymethyloxycarbonyl)-1-aryl-3-methyltriazenes **22**. Reagents: i. ClCH_2_OCOCl, CH_2_Cl_2_, triethylamine; ii. NaI, acetone; iii. RCO_2_Ag or RCOOH/Ag_2_CO_3_, acetone, tetrabutylammonium bromide [31].

Further enhancement of the chemical stability in isotonic phosphate buffer (80 min–7 days) was achieved by exploring the acyloxymethyl carbamate functionality, which was introduced as shown in Scheme 6. While this study demonstrated that the acyloxymethyl carbamate derivatives **22** provide an alternative prodrug system for the anticancer triazenes that bypass the need for oxidative demethylation, their low stability in human plasma (5 min–2 h) represents a significant drawback of this strategy.

Preliminary work on the non-specific prodrugs, which informed their chemical and enzymatic stability, was crucial for the design of specific methyltriazene prodrugs by exploiting enzymes and other conditions unique to the tumour microenvironment or specific targets. This review aims to highlight different preclinical approaches for targeted delivery of aryl methyltriazenes, the underpinning chemistry, kinetic studies and mechanisms of bioactivation. The rationale and synthesis of the different triazene hybrids will be discussed as well.

## 2. Specific Methyl Triazene Prodrugs

### 2.1. Targeting Tyrosinase

**Figure 3:**
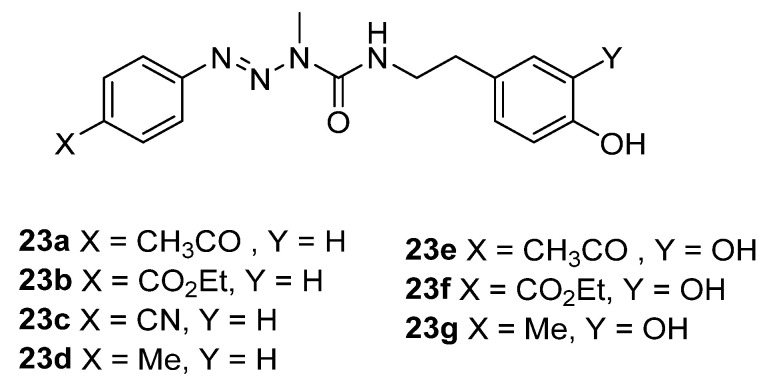
Dopamine- and tyramine-based triazene prodrugs [34].

Tyrosinase is overexpressed in malignant melanoma but largely absent in other cells, which makes it a potential target for prodrug development [32]. Interestingly, the degree of malignancy in melanoma has been shown to correlate with tyrosinase activity [33]. Tyrosinase is the main enzyme involved in the human pigmentation process, where it catalyses the hydroxylation of L-tyrosine to form dopaquinone, which then undergoes intramolecular Michael addition of the amino group to the quinone ring. This latter step specifically underlies the inspiration for the development of a tyrosinase-dependent prodrug strategy aimed at releasing nitrogen mustards specifically in melanocytes [32]. Building on previous work, Perry and co-workers developed a new class of targeted methyl triazenes by synthesising several prodrugs in which the methyl triazene was connected to dopamine or tyramine, as the tyrosine-sensitive unit, via a urea group (Figure 3) [34].

**Scheme 7:**
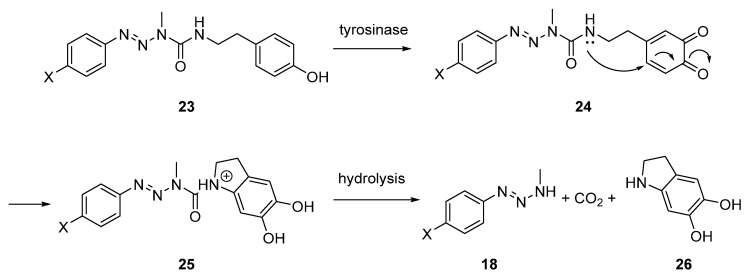
Proposed activation pathway for tyramine-based triazene prodrugs **23** triggered by tyrosinase via enzymatic oxidation [34].

These prodrugs were designed to be activated by tyrosinase by incorporating a phenol group susceptible to activation by the enzyme, with the release of the monomethyl triazene was envisaged to occur via the pathway depicted in Scheme 7. The process of cyclization renders the urea connection vulnerable to a hydrolytic attack in cyclic analogue **25**. Synthesis of the prodrugs was achieved via the reaction of 1-aryl-3-methyltriazenes with 4-nitrophenyl chloroformate, followed by nucleophilic attack of the amine group in dopamine or tyramine under basic conditions to generate the urea bond. The synthesised prodrugs not only showed excellent stability both in pH 7.4 buffer and in human plasma but also proved to be good substrates for tyrosinase. However, the release of the cytotoxic monomethyltriazene did not occur, as the reduced nucleophilicity of the modified amine nitrogen in triazenes **24** hindered the release of the alkylating triazene after enzymatic activation [34].

**Scheme 8:**
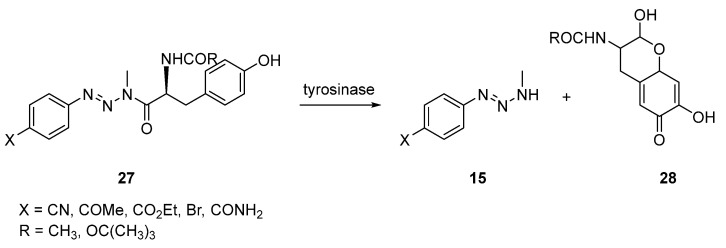
Representation of *N*-acyltyrosine-based triazene prodrugs **27** activated by enzymatic oxidation [35].

In order to provide effective release of the active metabolite in melanocytes, other tyrosinase-activated prodrugs were developed by the same group. These novel compounds (Scheme 8) included 3-aminoacyl triazenes **27** derived from *N*-acyltyrosine, an amino acid that is considered a good tyrosinase substrate. Interestingly, the authors reported the first microwave-assisted synthesis of triazenes as an alternative strategy to the traditional amide coupling reaction [35]. These triazenes **27** were successfully metabolised by tyrosinase with a concomitant release of monomethyltriazenes **15** and demonstrated excellent stability in a pH 7.4 buffer (approx. 40–72 h) as well as in human plasma (approx. 1.2–9.5 h), which suggests sufficient stability to reach the tumour cells intact.

**Scheme 9:**
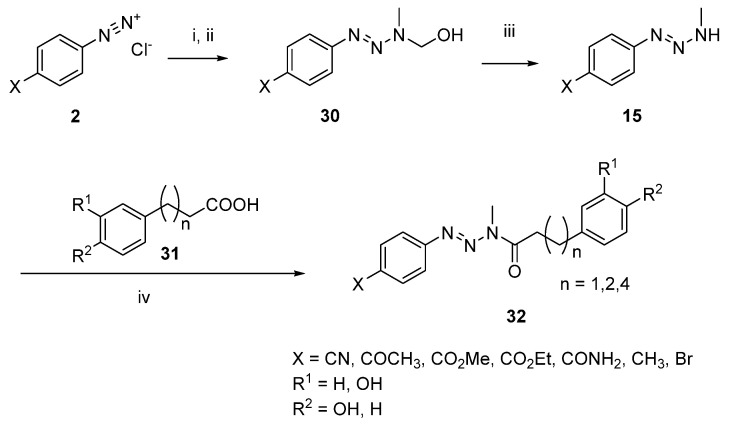
Synthetic route to acyltriazene **32.** Reagents and conditions: (i) NaOH until pH 7 and then formaldehyde; (ii) MeNH_2_; (iii) water, MeNH_2_; (iv) NaH, and DCC/DMAP or DMT/MM.

These novel prodrugs exhibited significant cytotoxicity, with the percentage of apoptotic/necrotic cells showing a direct correlation with the level of tyrosinase activity in melanoma cell lines, MNT-1 and B16F10. Compared to TMZ, these prodrugs showed increased specificity for tyrosinase-expressing cells, suggesting strong targeted therapeutic potential for melanoma treatment [35]. This work validated the advantage of an amide linker for the release of the active metabolite. Since then, evidence has shown that the α-amino group in **27** is not a requisite for tyrosinase affinity and activation, since novel triazene prodrugs **32**, derived from hydroxyphenylalkyl acids **31**, also demonstrated cytotoxicity in cell lines with a high level of expression of this enzyme [36]. Similar to *N*-acyltyrosine analogues, the most biologically active triazenes **32** exhibited greater cytotoxicity than the standard agent TMZ. The 3-Acyltriazenes **32** (Scheme 9) were accomplished using methyltriazene precursors **15**, which were derived from the corresponding hydroxymethyltriazenes **30** [13,37]. Depending on the carboxylic acid employed, different coupling reagents were used; however, the overall yields were low (<26%), likely due to the inherent instability of triazene **15**, which is prone to decomposition under the reaction conditions.

**Figure 4:**
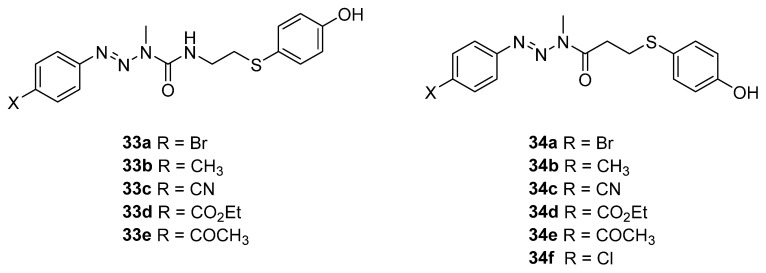
Triazene hybrid prodrugs based on sulfur analogues of tyrosine [38].

Melanoma-specific tyrosinase overexpression and catalytic activity has also been explored as a platform for the simultaneous delivery of two pharmacophores with antimelanoma activities [38]. Triazene hybrids **33** and **34** (Figure 4) were successfully metabolised by tyrosinase within 0.5 to 4 min, with kinetic studies of **33** confirmed the exhibition of a 2- to 4-fold higher affinity for mushroom tyrosinase than the natural L-tyrosine. Comparison of the reactivities of these hybrid prodrugs confirmed that urea derivatives **33** are more stable than amides **34** in PBS at 37 °C and in human plasma [38], which is in accordance with previous work by the same group [34,35,36]. In addition, since urea-derived compounds are poor substrates for plasma enzymes, premature hydrolysis is prevented, allowing the prodrug to remain intact in blood circulation, while arriving at the tumour site in high concentrations.

**Scheme 10:**
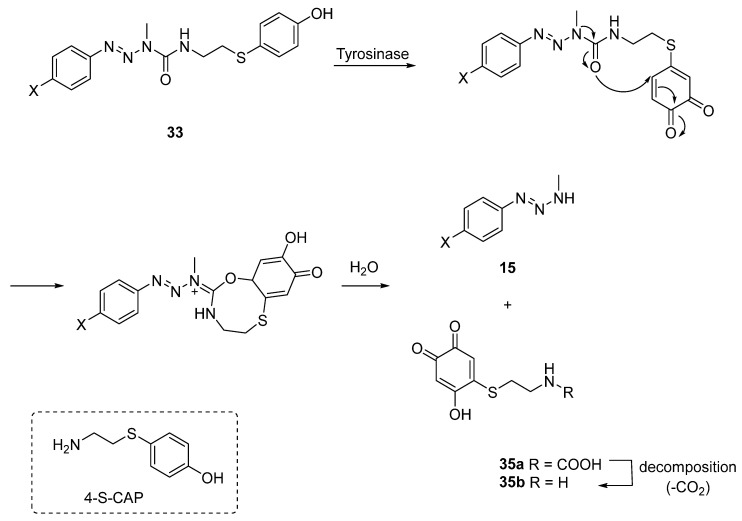
Tyrosinase-mediated metabolization of triazene hybrid **33** into monomethyltriazene [38].

Newer prodrugs **33**, unlike their urea-derived counterparts **23** [34], exhibited an increased and selective potency towards human melanoma cell line (MNT-1) and murine cell line (B16F10), without significant effect on healthy human keratinocyte cells (HaCaT). The MNT-1 melanoma cell line showed higher levels of tyrosinase than BF16F10 but still exhibited comparable cytotoxicity, suggesting higher sensitivity of the murine model to the alkylating methyltriazene. This selectivity most likely reflected their ability to cyclize once upon oxidation by tyrosinase, leading to the release of the active metabolite methyl triazene **15** and **35b**, an analogue of 4-*S*-cysteaminylphenol (4-*S*-CAP) (Scheme 10) [38]. 4-*S*-CAP is a substrate of melanoma tyrosinase that inhibits in vivo growth of malignant melanoma and causes depigmentation of dark skin [39,40]. Furthermore, evidence shows that the antitumour activity of **33** not only resulted from the conjugation of the two pharmacophores, but also depended on the type of linkage, since prodrugs containing an amide linker exhibited cytotoxic activity only against the A375 cell line. However, the low levels of tyrosinase expression in the A375 cell line suggests that the cytotoxicity of analogues **34** on the cell line might be due to the enzymatic instability of the amide bond in human plasma. Nonetheless, data obtained with urea-based analogues (**33**) confirms the superiority of a triazene hybrid strategy with its release of two biologically active agents. This also confirms how important the stability of the linkage between the promoeity and methyl triazene is. Compounds that were easily metabolised by tyrosinase or any other trigger but display low human plasma stability were ruled out since the alkylating unit would be released independently of the enzymatic process. Preliminary in vivo safety studies demonstrated absence of chemically induced toxicity in major organs and no adverse hepatic effects, an encouraging finding for the development of a tumour-targeted strategy [38].

### 2.2. Targeting Nitroreductase

GBM is the most common malignant primary brain tumour in adults, with an average survival of 12–15 months [41], and TMZ being the first-line treatment following diagnosis [42]. One key feature that advanced TMZ as a first-line therapy in the treatment of GBM is its ability to efficiently cross the blood–brain barrier, where its conversion to MTIC partially exploits the pH difference between the extracellular and intracellular environment of GBM tumours, allowing for oral administration [11,43]. Unfortunately, resistance to TMZ therapy is inevitable in glioma, resulting in disease relapse and poor prognosis [42].

**Scheme 11:**
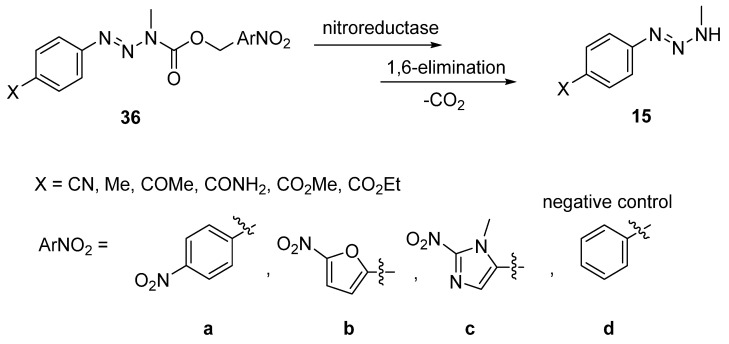
Nitroaromatic-based methyltriazene hypoxic-activated prodrugs **36**. ArNO_2_: bioreductive trigger.

The requirement for selective delivery of methyl diazonium in glioma, together with the recognition that, similar to other tumours, the low-oxygen environment of GBM [44] offers a promising therapeutic avenue for treatment, inspired Braga and co-workers to develop nitroaromatic-based methyltriazene derivatives **36** (Scheme 11) as hypoxia-activated prodrugs (HAP) [45]. It has previously been demonstrated that brain tumour samples could metabolise the experimental bioreductive drug, tirapazamine, to the two-electron reduction product, confirming these tumours as suitable targets for bioreductive therapy [46].

The synthesis of triazene-based HAP (**36**) proceeded via a carbamate bond formation between monomethyltriazene **15** and a bioreductive trigger, using either the appropriate nitroaryl alcohol activated with 1,1′-carbonyldiimidazole (CDI) or the corresponding nitroaryl chloroformate. A negative control lacking the nitro group (Scheme 11) was also synthesised [45]. The CNS multiparameter optimisation (CNS MPO) scores for these prodrugs ranged between 4.0 and 4.9, indicating that they have the potential to cross the blood–brain barrier (BBB) [47]. Amongst the synthesised prodrugs, those with the 5-nitrofurfuryl promoeity were the most extensively activated (within 1 min) by *Escherichia coli* nitroreductase (NTR) NfsB. However, subsequent studies revealed that a prodrug in this series was cytotoxic to human primary dermal fibroblasts, thereby excluding it as a viable targeted prodrug [45]. In contrast, three prodrugs from the 4-nitrobenzyl series (R = COMe, CO_2_Me and CO_2_Et) not only exhibited better selective cytotoxicity against glioblastoma cell lines (LN-229 and U87 MG) than the equivalent dose of TMZ, but also induced apoptosis in LN-229 cells, as well as senescence in U-87 cells under hypoxic conditions. However, the comparable cytotoxicity observed under both hypoxic and normoxic conditions highlights the need for further development as triazene-based HAP for glioblastoma treatment [45].

**Scheme 12:**
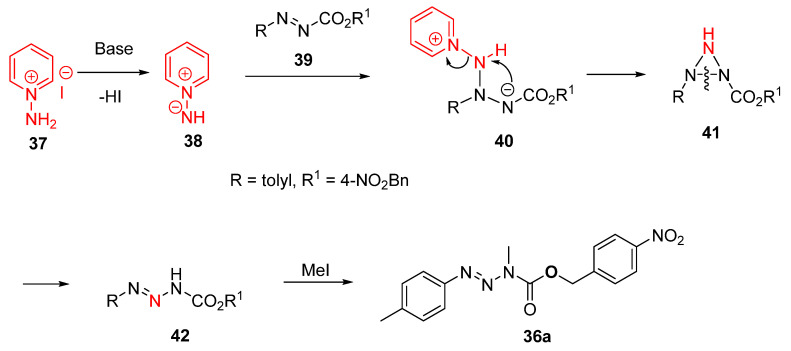
Proposed mechanism for the efficient insertion of nitrogen to prepare triazene carboxylates [48].

In a different study, prodrug **36a** (Scheme 12) was also obtained through the insertion of a single nitrogen atom into aryl diazene carboxylates **39** using *N*-amino pyridinium iodide **37** under basic conditions, followed by alkylation at the 3-position with methyl iodide. This recently developed approach offers mild, straightforward reaction conditions and accommodates a broad substrate scope. This novel methodology follows the aziridination-ring opening arrangement to insert a single nitrogen atom. The aziridination occurs through initial nucleophilic addition of the anion **38**, generated from the pyridinium salt, across the nitrogen double bond of the aryl diazene carboxylate **39** to generate a zwitterionic intermediate **40**. This intermediate undergoes intramolecular cyclization followed by pyridine elimination, leading to the formation of a triaziridine ring in **41**. Subsequent ring opening through ArN–NCO_2_R bond cleavage furnishes the aryl triazene carboxylate **42** (Scheme 12) [48]. However, this new method requires the preceding synthesis of the diazene, which provided a higher yield for the triazene-based HAP **36a** (85%) [48] compared to the 22% obtained when triazene **15** was reacted with 4-nitrobenzylchloroformate [45].

## 3. Triazene Hybrids

Drug hybridization is an important pharmacological strategy in drug design owing to the associated advantage of conferring diverse structural features and biological activities to chemical compounds. It also offers a sophisticated form of combination therapy, in which two or more different bioactive compounds can be fused covalently in a single molecule [49]. Such a hybrid drug construct can interact with multiple targets and/or mechanisms involved in proliferation, increasing anticancer drug potency as well as overcoming drug resistance [50]. This class of drugs is particularly important for methyltriazene prodrugs, where the methylation of DNA by the active metabolite methyl group triggers the activation of DNA damage repair (DDR) mechanisms, which are enabled by a plethora of signalling pathways in cancer. Furthermore, the release of an aromatic amine when a monomethyl triazene of type **MTIC** (**2**) undergoes hydrolysis in addition to methyl diazonium has inspired the development of triazene hybrids. In this approach, the methyltriazene is combined with another bioactive agent via the aromatic amine function, which is regenerated upon release of the active methyl diazonium.

Combi-triazenes are hybrids employed in the exploration of the tumour-targeting strategy designated as “combi-targeting”, where inhibitors of epidermal growth factor receptor (EGFR) tyrosine kinase (TK) are combined with DNA-damaging agents to irreversibly inhibit EGF-dependent tumorigenesis. The role and overexpression of EGFR in cancer, including in glioblastoma, is well documented [51], and this dysregulation drives tumorigenesis, making it a key drug target, with multiple EGFR inhibitors approved for clinical use [52]. Original reports of a “combi-triazene” molecule merged aminoquinazolines with a methyl triazene motif [53,54]. The design of these combi-triazenes was aimed at enhancing the potency of the alkylating agent as well as circumventing problems associated with the reversibility of EGFR inhibitors. The feasibility of this concept was well demonstrated with two combi-triazene prototypes, SMA41 (**45a**) and BJ200 (**45b**), both of which were small enough to bind the EGFR ATP binding site and behave as a competitive enzyme inhibitor while also producing DNA damage. An example of this is seen in EGFR-overexpressing A431 cells, where antiproliferative studies of SMA41 and BJ200 confirmed their ability to both damage DNA and block EGFR autophosphorylation [53,54].

**Scheme 13:**
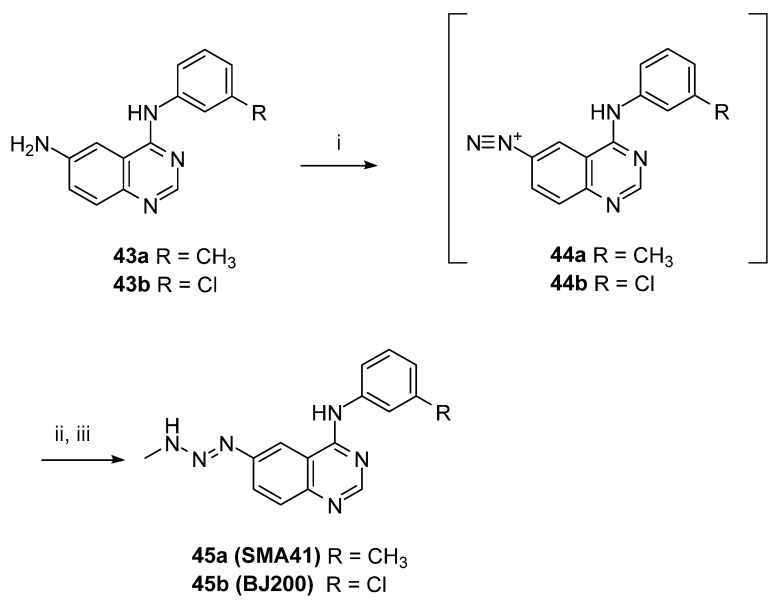
Synthesis of combi-triazenes SMA41 (**45a**) and BJ200 (**45b**). Reagents and conditions: (i) NOBF_4_, MeCN, 0 °C; (ii) aq. CH_3_NH_2_; (iii) K_2_CO_3_.

**Scheme 14:**
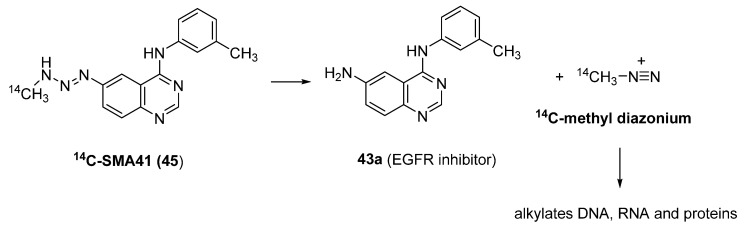
Degradation of ^14^C-labelled combi-triazene SMA41.

Synthesis of quinazolinyl triazene **45a** (SMA41, Scheme 13) produced good yields. Treatment of aminoquinazoline **43a** [55] with nitrosonium tetrafluoroborate in cold acetonitrile provided the intermediate diazonium salt **44a**, which in turn was reacted with aqueous methylamine in situ, followed by raising the pH of the resulting mixture with potassium carbonate [53]. Alternatively, using ^14^C-methylamine hydrochloride as the amine to react with intermediate **44a** led to the radiosynthesis of ^14^C-labelled SMA41 **45** [56]. Biodistribution studies with this ^14^C-labelled SMA41 allowed the tracking of the released ^14^C-mehyl diazonium. It was demonstrated that the ^14^C-methyl group was distributed throughout DNA, RNA and mainly protein, which showed that SMA41 diffused into the cells followed by decomposition into 6-amino-4-anilinoquinazoline **43a** and methyl diazonium that non-specifically alkylates RNA, proteins and nuclear DNA (Scheme 14) [57]. Previous analysis demonstrated that in cell culture media, SMA41 (**45a**) could be converted to quinazoline diamine **43a** in an 80% yield with a half-life of approximately 34 min., as determined by UV analysis [58].

**Figure 5:**
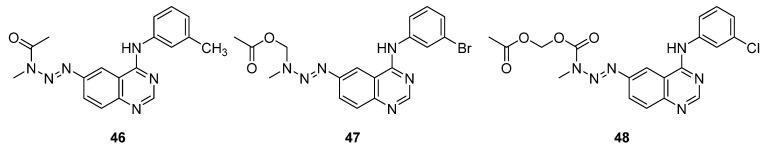
Examples of cascade-release prodrugs of EGFR-based combi-triazenes [59,66].

**Scheme 15:**
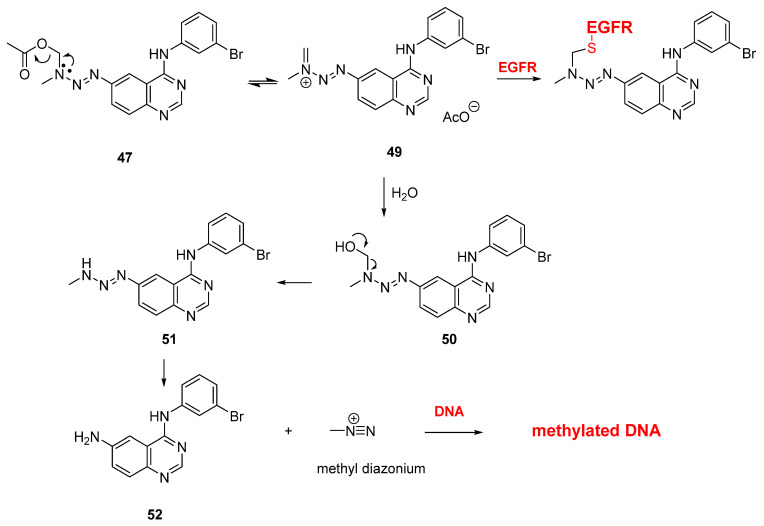
Proposed cascade degradation of prodrug **47** and its mechanism of action [61].

To enhance the EGFR inhibitory potency and stability of these combi-triazenes, a new strategy termed “cascade release” (CR) was explored, with the aim of masking the combi-molecule as a prodrug (Figure 5) intended to simultaneously release both antitumour agents (EGFR inhibitor and DNA-alkylating agent) by hydrolytic activation. Prodrug **47** showed similar EGFR binding affinity as the parental combi-triazene, since the acylation had no effect on the π-extended system of 1,2,3-triazene. However, when the acylation is directly in the triazene, as in prodrug **46**, the binding affinity to the ATP site of EGFR was 3-fold lower [59]. Structure–activity relationship (SAR) studies of the quinazolines showed dependence on the electronic character of the substituents at the 6-position, with electron-donating groups producing an increase in affinity while electron-withdrawing groups produce a deleterious effect [55,60]. Prodrug **46,** which was prepared in poor yield (18%) by reacting combi-triazene **45a** with acetyl chloride in chloroform, was not suitable for the CR strategy as it displayed high hydrolytic stability under physiological conditions (serum-containing media at 37 °C), even after 48 h. An alternative prodrug **47** was much less stable with a half-life of 42 min. in serum-containing media [59]. Furthermore, while prodrug **47** had no effect on chemical stability, it induced significant levels of DNA strand breaks with 100-fold greater potency than its single-targeted DNA-damaging counterpart TMZ [61]. Jean-Claude and co-workers postulated that the pronounced antiproliferative potency of **47** was due to its ability to simultaneously damage DNA and irreversibly inhibit EGFR activity through the multistep degradation cascade of **47**, which generated two electrophiles, an iminium ion (**49**) and methyl diazonium (Scheme 15). Indeed, acetoxymethyltriazene **47** acted as an irreversible inhibitor of EGFR, while the corresponding quinazoline **52** induced only reversible inhibition [61]. It must be noted that combi-triazenes **45a** and **45b** also acted as irreversible inhibitors of EGFR, but the antiproliferative potency only increased 8-fold and 11-fold, respectively, against A431 cells in comparison to TMZ [62]. As previously stated, studies with radiolabeled **^14^C-SMA41** showed that protein was also methylated in addition to DNA and RNA, which in itself might have contributed to the irreversibility observed with compounds **45**.

Furthermore, A431 cells are phenotypically characterised by their overexpression of EGFR, as well as the expression of *O*^6^-methylguanine methyl transferase (O^6^-MGMT), a DNA repair enzyme that repairs the *O*^6^-alkylguanosine DNA adduct and induces resistance to methylating agents. The chemical feature of prodrug **47** conferred superior antiproliferative activity in a cell line in which a classical methylating agent of the same class did not show any detectable activity. However, the half-life of **47** is comparable to the half-lives of acetoxymethyltriazenes **14** [24,63], which are rapidly converted to the corresponding methyl triazenes and parental combi-triazene (*t*_1/2_ = 45 min). Further attempts to increase the bioavailability of combi-molecules led to the design of the corresponding 3,3-dimethyltriazene prodrugs intended to be metabolically activated. However, this type of molecule failed to generate adequate levels of monoalkyltriazenes in vivo as the quinazoline moiety might have blocked CYP activation [64]. To remedy this, the combi-triazene **45b** was masked with carbamates featuring different leaving groups (vinyl, chloroalkyl, acetoxymethyl and *p*-nitrophenyl carbamates) to modulate the hydrolysis following addition on the carbonyl moiety of the N3-carbamyltriazene **48** (Figure 5) [65]. This study conclusively demonstrated that combi-molecules stabilised by attaching a carbamate to the N3 of the triazene chain achieved greater stability compared to the previously reported acetoxymethyl-N3 strategy [59,61]. More importantly, it was shown that stability could be achieved without any significant loss in the binary EGFR-DNA targeting properties of the identified lead molecule **48** (Figure 5). Synthesis of **48** and related carbamates was accomplished using similar procedures to the acyloxymethyl prodrugs **22** [31].

**Scheme 16:**
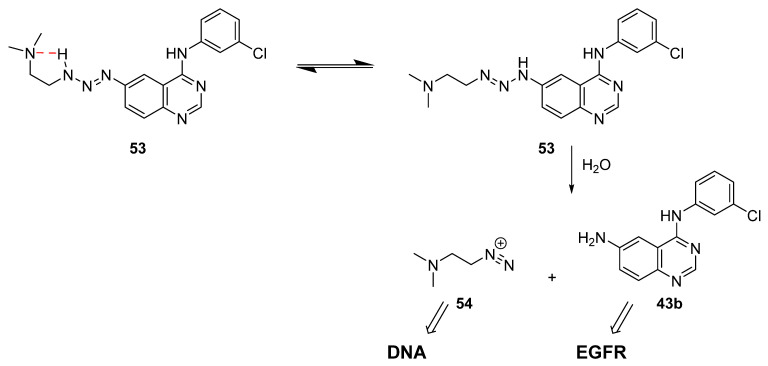
Hydrolysis and binary targeting of combi-triazene **53**.

SMA41 (**45a**) was the first combi-triazene studied in vivo, but it showed only moderate activity in a preclinical tumour xenograft model (A431; carcinoma of the vulva). This was attributed to its poor water solubility, lack of sensitivity of A431 cells that express O^6^-MGMT, and a very short half-life [67]. A new quinazolinotriazene (**53**) (Scheme 16) was developed, containing a polar *N*,*N*-dimethylaminoethyl group attached to the N3 of the triazene, where it could serve both as a water-soluble and more potent alkylating moiety. The goal was to generate *N*,*N*-dimethylaminoethylguanine adducts, which were anticipated to serve as poorer substrates for MGMT than *O*^6^-methylguanosine (*O*^6^-MeG) (Scheme 1), resulting in higher potency [68]. Combi-triazene **53** was more stable (*t*_1/2_ = 108 min.) than SMA41, and the higher stability of this class of triazenes may be due to their ability to form intramolecular hydrogen bonding (Scheme 16) that stabilises the conjugated tautomer. Compound **53** showed 5-fold stronger EGFR tyrosine kinase inhibitory activity than SMA41, and marked binary targeting properties involving the simultaneous inhibition of EGFR phosphorylation in addition to DNA damage induction. Contrary to **45b** and SMA41, compound **53** triggered substantial levels of apoptosis. Furthermore, **53** showed significantly greater antitumour activity (*p* < 0.05) than SMA41 in the human MDA-MB-468 breast cancer xenograft model. The results indicated that the appendage of *N*,*N*-dimethylaminoethyl to combi-triazenes could offer an alternative solution to the reduced water solubility and lack of potency associated with monofunctional combi-triazenes against resistant tumours [68].

**Scheme 17:**
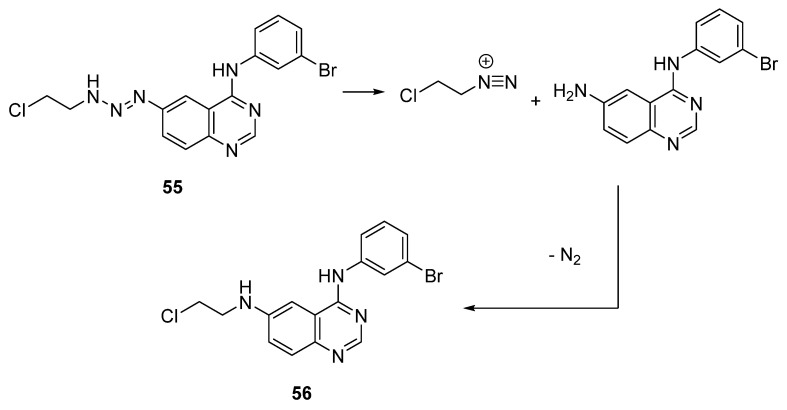
Proposed mechanism for the formation of combi-molecule **56** [69].

**Figure 6:**
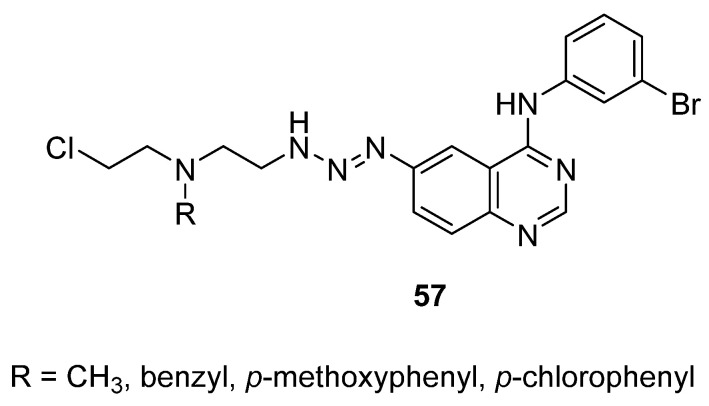
Chemical structure of combi-triazene **57** [72].

Interestingly, Jean-Claude and co-workers designed an alternative combi-triazene **55** (Scheme 17) with a chloroethyl group attached to the N3 of the triazene [69]. Chloroethyl is the tetrazine appendage in the preclinical MTZ (Figure 1), where the formation of the active chloroethyl diazonium species induces *N*1-guanine–*N*3-cytosine strand crosslinks, leading to cell death [70]. Synthesis of combi-triazene **55** was attempted by reacting aryl diazonium with chloroethylamine in the presence of triethylamines. However, under the basic conditions, **55** was found to be unstable and rearranged to form **56** following the loss of a nitrogen molecule [69]. Combi-molecule **56**, combining an EGFR inhibitor with a ‘half-mustard’ moiety, was found to be a potent EGFR inhibitor and exhibited fluorescence properties that permitted the quantitation of subcellular uptake by flow cytometry. The fluorescence intensities increased with increasing levels of EGFR in a panel of isogenic and established cell lines [69]. It was demonstrated that **56** was the first prototype of a combi-molecule capable of generating the binary EGFR/DNA targeting activity without the requirement for hydrolytic cleavage [71]. Further, to circumvent the problems associated with **55**, the same authors designed combi-triazene **57** (Figure 6), which contains a chloroethylaminoethyl group that conferred a second alkylating function to the released alkyldiazonium species [72].

**Figure 7:**
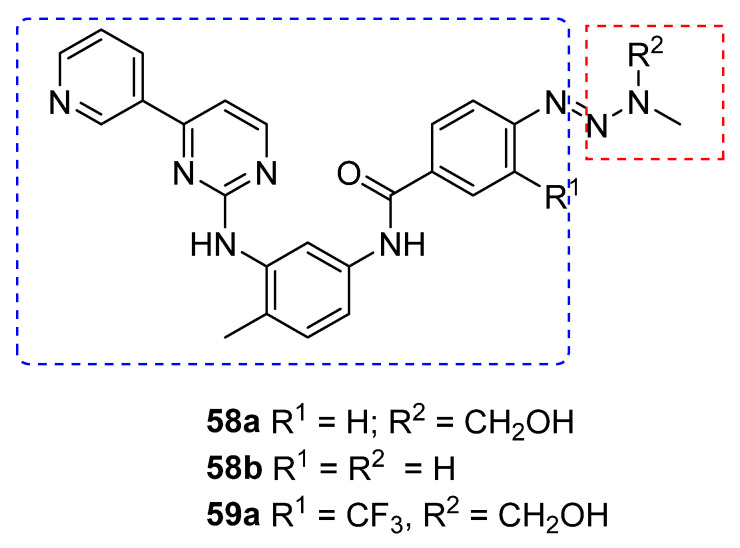
Chemical structure of combi-triazenes **58a** and **59a** for dual targeting of Bcr-Abl and DNA [73]. Blue box: Bcr-Abl-targeting moiety; red box: DNA-alkylating moiety.

Within the same combi-triazene approach, hybrid **58a** (Figure 7), comprising a monomethyl triazene conjugated to a 2-phenylaminopyrimidopyridine moiety, was engineered for the dual targeting of Bcr-Abl and DNA [73]. Bcr-Abl is a constitutively activated tyrosine kinase implicated in chronic myelogenous leukaemia (CML). With the demonstration that imatinib, a Bcr-Abl inhibitor, sensitised Bcr-Abl-expressing cells to cytotoxic DNA-damaging agents by depleting the antiapoptotic properties associated with the Bcr-Abl pathway, there was an impetus to design a combi-triazene hybrid [74,75]. In cell culture media, **58a** was hydrolysed into **58b** with a half-life of 27 min. In leukaemia cells expressing varied levels of Bcr-Abl, **58a** was consistently more potent than TMZ, and this was attributed to its ability to simultaneously block Bcr-Abl and related DNA repair activity, while inducing significant DNA lesions in Bcr-Abl-expressing cells [73]. Nonetheless, **58a** exhibited weak Bcr–Abl inhibitory activity, which led to the design of **59a** (Figure 7), incorporating a trifluoromethyl-substituted benzamide moiety envisioned to enhance Bcr–Abl potency [76].

**Figure 8:**
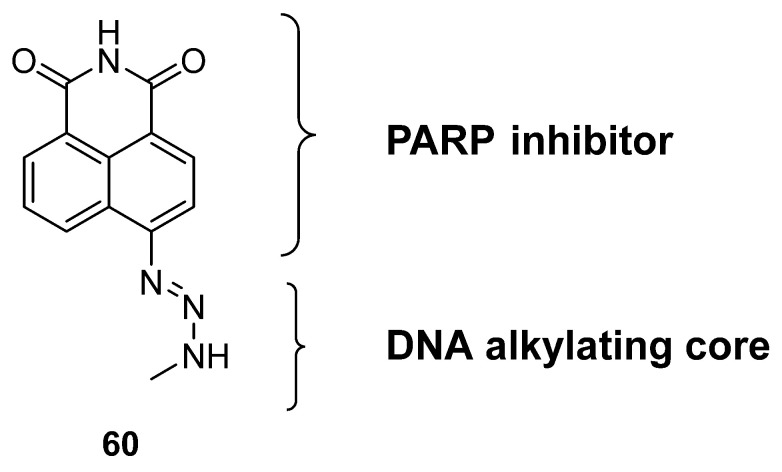
Chemical structure of combi-triazene for dual targeting of PARP and DNA [79].

Other combi-triazenes were designed to disrupt the DDR pathways responsible for the repair of DNA adducts generated by the methylating agents. The majority of these adducts are processed through poly(ADP-ribose) polymerase (PARP)-dependent base excision repair machinery. Indeed, several ongoing clinical trials are exploring the combination of PARP inhibitors with TMZ [77], which motivated the design of the molecular hybrid **60** (Figure 8). Compound **60** consisted of the conjugation of the PARP inhibitor 4-amino-1,8-naphthalimide (ANI) to a monomethyltriazene, with synthesis accomplished via diazotization, as described in Scheme 3A, and its ^15^N and ^13^C-labelled form was also synthesised for characterisation purposes [78,79]. Biological studies on **60** demonstrated higher levels of DNA damage than TMZ and showed strong potency in both BRCA1/2 wild-type and mutated cells, with a 6-fold selectivity for the mutants. Since acquired resistance to PARP inhibitors is mediated by the reactivation of wild-type BRCA1/2 [80], this suggests that the potentiation observed with PARP inhibition is attributable to the methyl alkylating unit. Furthermore, **60** was 65–303-fold more potent than TMZ, 4–63-fold than ANI alone, and 3–47-fold more than their corresponding equimolar combinations. Its potency was independent of MGMT expression, which suggested that this combi-molecular approach directed at blocking PARP and damaging DNA can lead to single molecules with selective and enhanced potency against BRCA1/2 mutant cells, and with an activity independent of MGMT, the major predictive biomarker for resistance to TMZ [79].

**Scheme 18:**
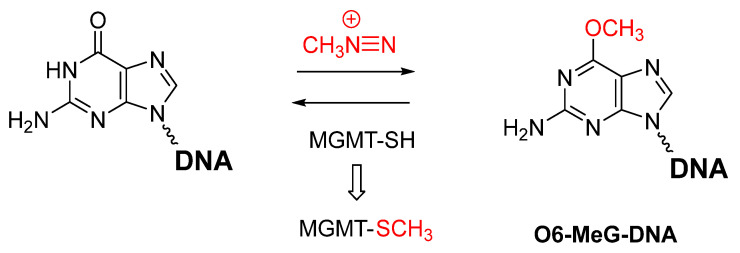
Methylation of DNA and MGMT-catalysed demethylation of guanosine residues [81].

**Scheme 19:**
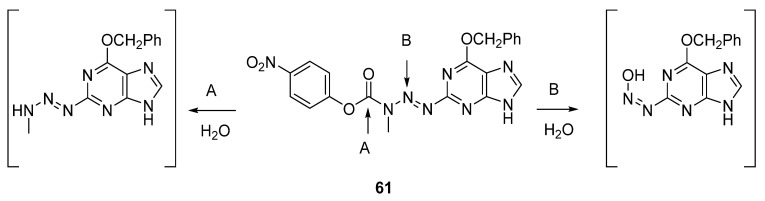
Chemical structure of triazene hybrid **61** for dual targeting of MGMT and DNA [82].

**Scheme 20:**
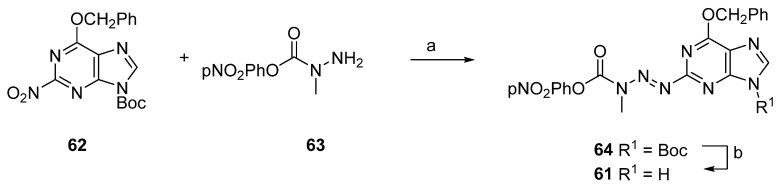
Synthetic route to triazene hybrid **61** [82]. Reagents and conditions: (a) acetic acid/CH_2_Cl_2_ 1:3, rt, 18 h; (b) CH_3_CN/water, reflux, 30 min.

Furthermore, with evidence of the role of MGMT in DNA damage repair and chemoresistance to alkylating agents, including TMZ and DTIC, the covalent combination of monomethyltriazene with *O*^6^-methylguanine-DNA methyl transferase (MGMT) inhibitors has been developed [81]. This is particularly important since MGMT demethylates the DNA-guanosine residues (Scheme 18) that account for the therapeutic effect of the methyl diazonium. Therefore, MGMT has been considered a promising target to enhance the therapeutic efficacy of methyl diazonium and evade resistance. This motivated the design of hybrid **61** (Scheme 19), which involved the direct conjugation of the monomethyl triazene to the aromatic amine of the *O*^6^-benzyl guanine, a known MGMT inhibitor [82]. Like TMZ and DTIC, the highly electron-deficient triazene **61** showed an optimal hydrolysis rate, while the carbamate group favoured the fragmentation route via hydrolysis at the carbonyl of the carbamoyl group (path A). In addition, the instability of the purinyl diazonium compounds precluded the application of the classical method of acylated triazene preparation in these analogues. Synthesis of **61** and related analogues was achieved via condensation of heterocyclic nitroso derivative **62** with hydrazide **63** (Scheme 20), which is similar to the conditions employed for the synthesis of the triazene moiety of TMZ [83], and particularly well suited to the preparation of electron-deficient triazenes [84] Chemosensitivity studies in the NCI 60 human tumour cell line panel identified hybrid **61** as the most active dual targeting agent for MGMT and DNA, exhibiting an IC_50_ of 10 µM, compared to 100 µM for TMZ [82]. Further studies would benefit from expression studies confirming the presence or absence of MGMT to correlate the higher cytotoxicity with simultaneous DNA alkylation and MGMT inhibitor mechanisms. Since MGMT is responsible for temozolomide resistance in GBM, further studies should be performed in a temozolomide-resistant cancer cell line with high level of MGMT expression (U373M) to confirm the benefit of a triazene hybrid conjugated to an MGMT inhibitor.

Along similar lines, to overcome the unsatisfactory therapeutic efficacy of TMZ in GBM, a new triazene hybrid was designed for targeted cancer therapy, which involved the combination of the aryl methyltriazene with valproic acid (VPA). VPA is an antiepileptic drug and histone deacetylases (HDAC) inhibitor used to control seizures in patients with brain tumours [85]. Further evidence that the combination of VPA and TMZ produced potentiated antitumour effects in TMZ-resistant glioma cells [86], as well as the amenability of the carboxylic group of the VPA to conjugation at the N3-position of the triazene, supported the development of hybrid **65** (Scheme 10) [87]. The synthesis of hybrid **65** was carried out in the same manner as for *N*-acyltriazene **32** (Scheme 9). Preclinical evaluation showed that hybrid **65** exhibited a stronger antiproliferative effect on GL261 glioma cells than TMZ, with cytotoxic activity predominantly targeting cancer cells, while producing a markedly lower impact on normal astrocytes. Moreover, not only was an enhanced autophagic activity observed with **65**, which might have contributed to its increased cytotoxicity, but it also induced an alteration in the morphology of GL261 cells, producing a non-polar, rounded phenotype that impaired their migratory capacity. Notably, unlike TMZ, cells exposed to **65** maintained drug resistance levels close to control conditions [87].

**Figure 9:**
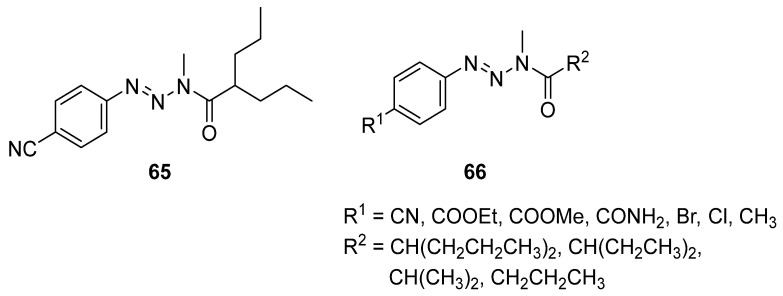
Chemical structures of triazene-valproic acid **65** and related analogues **66** [87,88].

SAR and mechanistic studies of a series of *N*-acyltriazenes **66** (Figure 9) and their anti-glioma activity [88], showed that these hybrid compounds incorporated triazene moieties bearing various substituents along with HDAC inhibitor-related short-chain fatty acids such as valproic and butyric acids, including hybrid **65** previously studied. Similar to the *N*-acyltriazenes **32** and **65**, these analogues **66** were isolated only in moderate yields (approximately 23–25%), even when HOBt was used to promote the coupling rearrangement process. Compared to the lead molecule **65**, hybrid agent **66** containing an aryl carboxamide (R^1^) triazene conjugated to VPA (R^2^) was found to be the most potent in this series, showing selective targeting toward GL261 glioma cells and an effective decrease in invasive cell properties. In contrast to TMZ, which undergoes hydrolysis to release the alkylating metabolite monomethyltriazene, hybrids **66** showed enhanced chemical and metabolic stability. Moreover, the full conjugates **66** exhibited both low DNA-alkylating potential and weak HDAC inhibition, indicating that their antiproliferative activity depended on the release of *both* anticancer agents [88]. These findings support valproate triazene hybrids as a new class of targeted agents, offering clear chemotherapeutic advantages over TMZ and demonstrating strong potential for application in glioblastoma treatment [87,88].

**Scheme 21:**
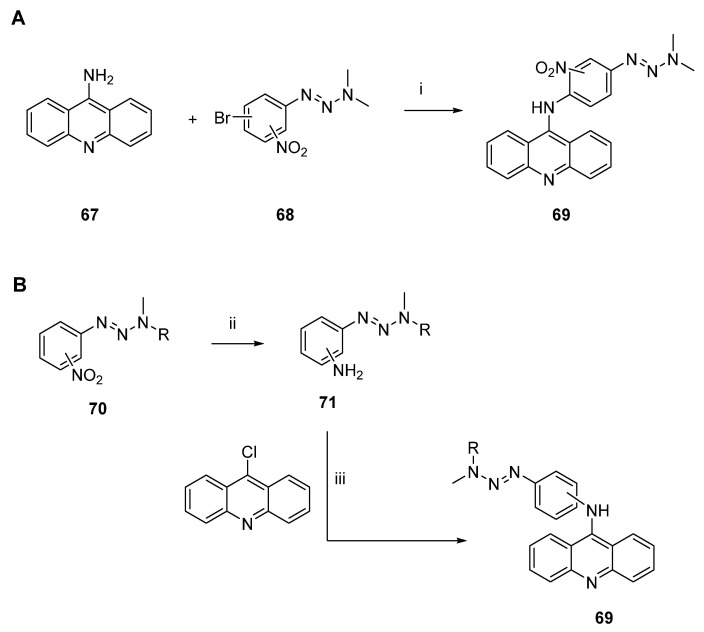
Synthesis of triazene 9-AnA chimaeras **69** by nucleophilic substitution using either aminoacridine (**A**) or chloroacridine (**B**) [89]. Reagents and conditions: i. Cs_2_CO_3_, DMF, 90 °C, 2 h; ii.10% Pd/C, H_2_ (1 atm), EtOH, rt, 3 h; iii. NaH, DMF, 0 °C to rt, 12 h.

**Figure 10:**
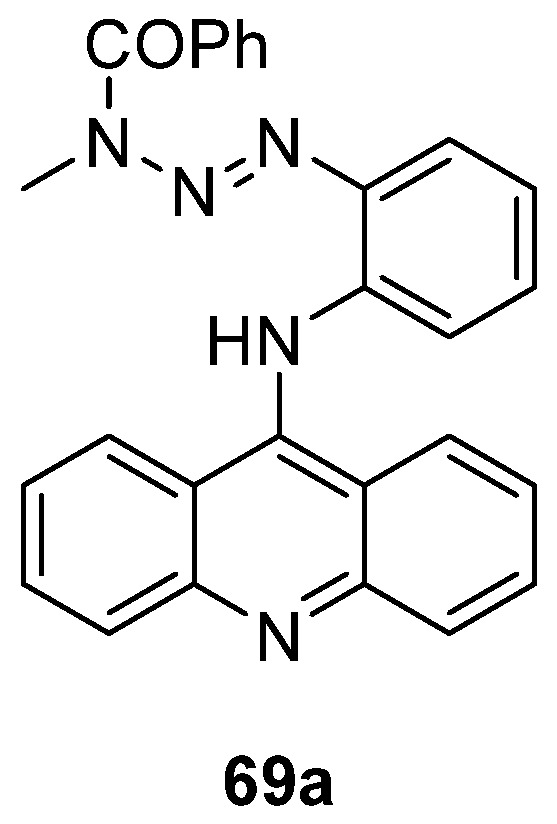
Lead methyl triazene 9-AnA chimaera.

Other hybrids have been developed by other researchers. Gellerman and co-workers called these molecular chimaeras, with their group developing a wide range of structurally different combinations of methyl triazenes and various pharmacophores. They investigated the dual action of methyl triazene chimaeras containing the DNA-intercalating 9-anilinoacridine [89], which has served as a widely utilised structural core for hybrid drug candidates over the last three and a half decades [90]. The planar structure of the acridine associated with the presence of both hydrogen acceptor and donor groups allows it to intercalate the DNA and bind to the minor groove, stabilising the DNA-drug complex [91,92]. However, these have also been impeded by several clinical drawbacks. One of these is the fast deactivation of amsacrine, employed in the treatment of acute lymphoblastic leukaemia, by glutathione-associated drug resistance mechanisms. This limitation prompted the design of new-generation analogues and ultimately led to the development of 9-anilinoacridines (9-AnA) and new triazene-acridine chimaeras. Syntheses of nine bifunctional anticancer agents have been straightforwardly achieved via a nucleophilic aromatic substitution (Scheme 21) reaction, using readily accessible 9-AnAs and methyl triazene structural building blocks [89]. Preliminary antiproliferative assays identified chimaera **69a** (Figure 10), which displayed potent cell-growth inhibition with IC_50_ values of 2.9 μM and 0.8 μM against H1299 (NSCLC) and WM-266-4 (human metastatic melanoma) cells, respectively. Further studies demonstrated that this antiproliferative activity arises from two independent mechanisms: DNA alkylation and topoisomerase II inhibition [89].

**Figure 11:**
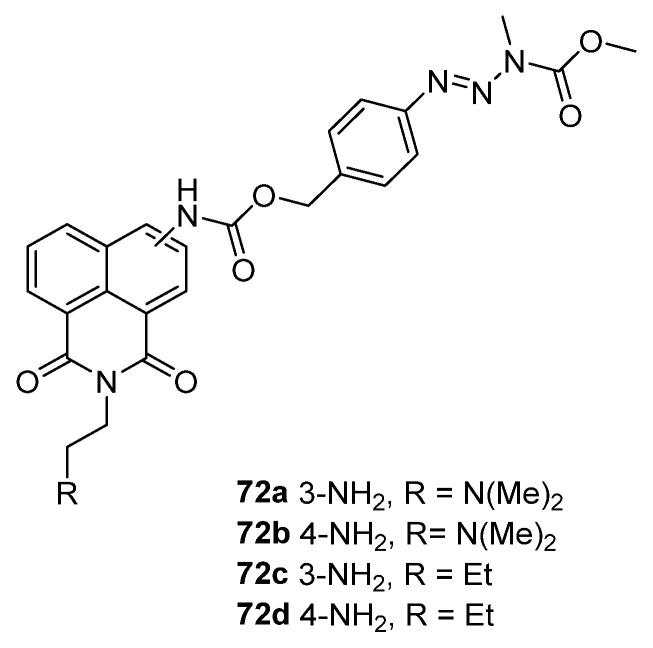
Chemical structures of aminafidazene hybrids **72** [94].

**Scheme 22:**
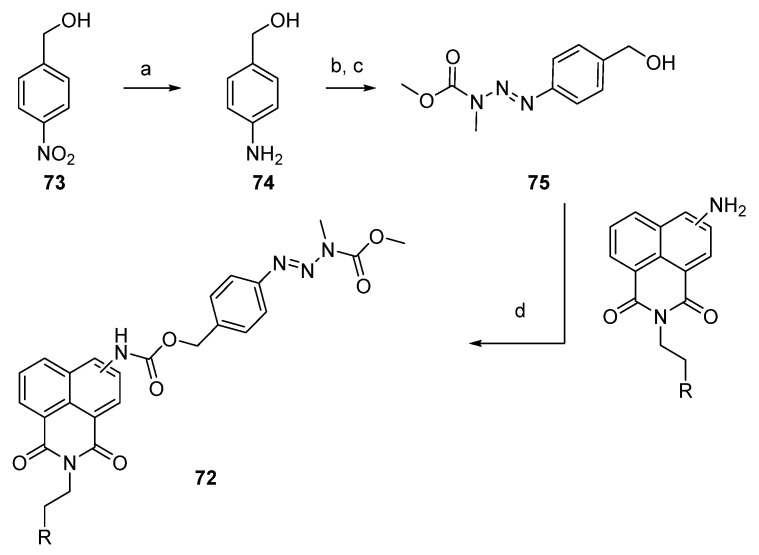
Synthesis of monomethyl triazene hybrids **72** [94]. Reagents and conditions: (a) Pd/C, H_2_ (1 atm), MeOH, rt, 12 h; (b) i. NaNO_2_/HCl, 0 °C, 45 min; ii. MeNH_2_; (c) ClCO_2_Me, CH_2_Cl_2_, DIPEA, 0 °C to rt, 6 h; (d) i. triphosgene, DIPEA, CH_2_Cl_2_, 0 °C; ii. azene (**75**)**,** CH_2_Cl_2_, rt, 24 h.

The identification of the effectiveness of the potent molecular chimaera CM358, formed by conjugating amonafide with chlorambucil, against human metastatic melanoma [93], motivated the development of related hybrids that instead exploited a methyl-triazene core, establishing an additional DNA-reactive triazene-hybrid platform [94]. Like acridines, amonafide is a topoisomerase II inhibitor which triggers a double-strand break (DSB) and cell death. These new chimaeras (aminafidazene) **72a** and **72b** (Figure 11) consisted of amonafide, or its 4-amino isomers, and the methyl triazene moiety, protected with a carbamate group and tethered through a self-immolative 4-aminobenzyl carbamate linker. Model chimeric compounds **72c** and **72d**, which lack the dimethylamino group, were also prepared to allow differentiation of the biological effects of the monomethyl-triazene core from those of the *N*-dimethylaminoethyl arm. The synthesis of these hybrids began with the synthesis of azene **75** (Scheme 19), comprising a monomethyl-triazene moiety protected and stabilised by a methyl carbamate group, and obtained through a well-known transformation which started with diazotization of 4-aminobenzyl alcohol **74,** followed by condensation with 40% aqueous methyl amine. Monomethyl triazene was quickly purified by extractive work-up and immediately reacted with methyl chloroformate to afford the carbamate azene **75** in good yield (ca. 60%). Then, hybrids **72** were synthesised in moderate to good yields (47–71%) in two sequential steps. First, the amino naphthalimides were transformed into the respective reactive isocyanates followed by addition of azene **75** (Scheme 22). Exposure of the HTC116 cancer cell line to the chimeric molecules **72a** and **72b** induced markedly increased levels of DSBs, accompanied by delayed repair kinetics compared with cells treated with amonafide or monomethyl-triazene alone. Conversely, incorporation of the methyl-triazene unit into the non-intercalating amonafide analogues **72c** and **72d** did not produce these effects. Consistent with the elevated levels of DSBs, compounds **72a** and **72b** exhibited significantly stronger antiproliferative activity in HCT116 cancers than either amonafide or azene **75**. The red-shift in fluorescence observed upon release of the amino-naphthalimide core enabled real-time monitoring of prodrug activation, confirming that the chimaera selectively accumulated and became activated at tumour sites. In a xenograft model in vivo, this targeted activation translated into significantly enhanced tumour-suppressive efficacy compared with amonafide. These findings suggest that targeting methyl-alkylating groups to the vicinity of DSB sites may represent a promising strategy for improving the anticancer potency of topoisomerase II inhibitors [94].

**Scheme 23:**
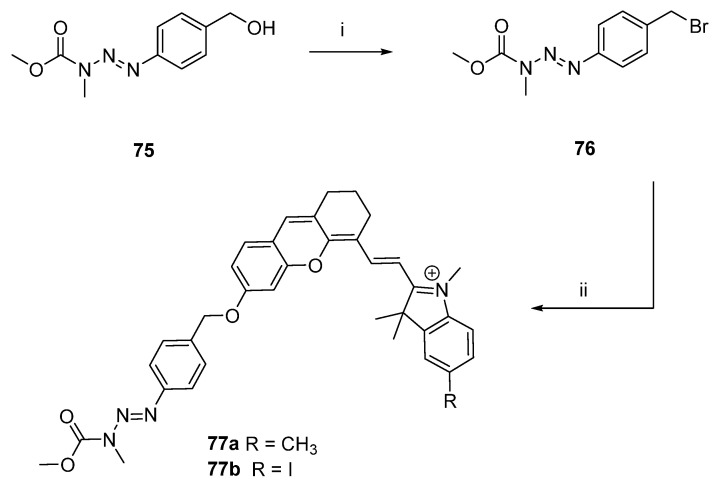
Synthesis of activatable chimaeras **77** [95]. Reagents and conditions: (i) PBr_3_, CH_2_Cl_2_, 0 °C, 30 min; (ii) xCy or I-xCy, K_2_CO_3_, CH_3_CN, rt.

Another theranostic chimaera was developed, which consisted of the DNA-methylating methyl moiety conjugated to a fluorogenic xanthene–cyanine dye as a photosensitizer [95]. The activatable near-infrared (NIR) dye XCy has been previously explored for fluorescence drug delivery monitoring [96] and photodynamic therapy (PDT) applications [97]. The photosensitizer component of this molecular hybrid provides a long-wavelength fluorescent signal, which was exploited for monitoring drug delivery and accumulation for photodynamic therapy (PDT). Synthesis of activatable fluorescent chimaeras **77a** and **77b** started with azene **75**, which contained the monomethyl triazene moiety protected and stabilised by methyl carbamate, as reported above [94]. Furthermore, **75** was transformed into its brominated counterpart **76** in very good yield (68%), followed by nucleophilic substitution by the phenol of XCy and I-XCy (Scheme 23). These chimaeras were obtained in good yields (47–58%) after preparative HPLC purification [95].

**Scheme 24:**
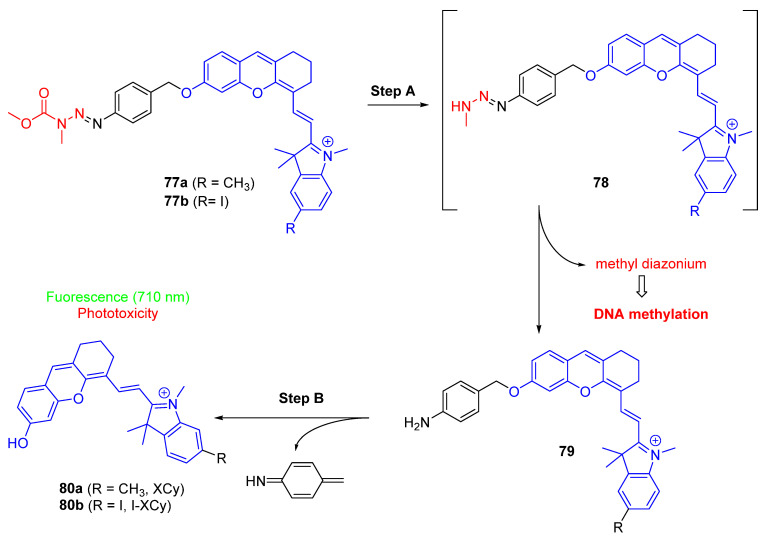
Proposed mechanism for fluorescence following activation of photosensitizer and DNA methylating agent. Step A. Carbamate bond cleavage; Step B. Self-immolative cleavage [95].

The suggested mechanism of action of the chimaeras **77** is shown in Scheme 24. These chimaeras are neither phototoxic nor fluorescent because the chromophore exists in the “off” form with the protected hydroxyl function [96]. Upon the environmentally mediated cleavage of the biodegradable carbamate group [94], the highly unstable monomethyl triazene **78** is formed, which is spontaneously cleaved to produce the highly reactive methyl carbocation causing the DNA methylation. In the next step, the ether bond of **79** is self-immolatively cleaved, yielding the “on” form of the xanthene–cyanine **XCy** (**80a**) and iodo-xanthene–cyanine **I-XCy** (**80b**) chromophore. The **XCy** chromophore generates highly cytotoxic species under NIR light excitation and, in addition, provided an NIR fluorescence signal. Thus, the free **XCy** dye acted both as the NIR photosensitizer and the fluorescent reporter signalling the drug activation events. Preliminary antiproliferative assays demonstrated that the developed chimaeras exhibited higher antitumour activity in breast cancer cells (MDA-MB-238) upon NIR light irradiation compared to their structural constituents, the xanthene-cyanine photosensitizer and monomethyl triazene components [95].

**Figure 12:**
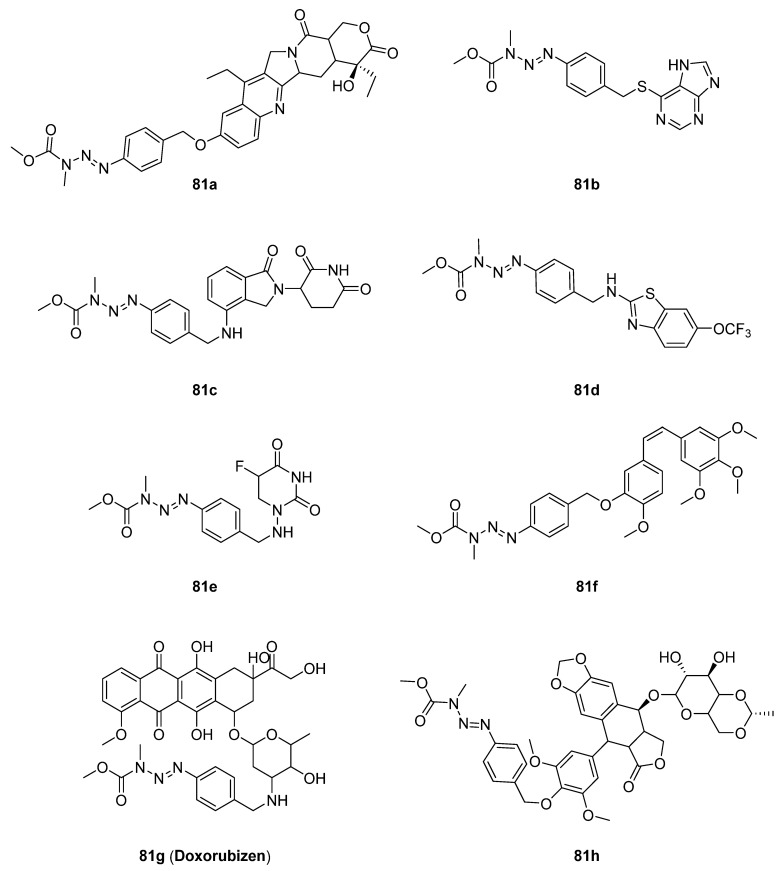
Chemical structures of chimaeras **81a**–**81h** [98]. Combination of carbamate methyl triazene with SN38 (**81a**); 6-mercaptopurin (**81b**); lenalidomide (**81c**); riluzole (**81d**); 5-fluorouracil (**81e**); combretastatin (**81f**); doxorubicin (**81g**) (Doxorubizen); Etoposide (**81h**).

More recently, conjugation of the carbamate azenebromide **76** with different FDA-approved anticancer drugs produced eight different chimaeras (Figure 12), featuring different combinations of two pharmacophores, which following investigation in various leukaemia cell lines, led to the identification of a potent chimaera, doxorubizen (**81g**), a sequel of the known DNA intercalator and topoisomerase II (Topo-II) inhibitor doxorubicin (Dox) and azene [98]. Molecular docking and molecular dynamic simulations identified doxorubizen as a more potent Topo-II inhibitor than Dox, owing to its ability to bind within the major groove of DNA. In addition, the monomethyl triazene group is optimally positioned through intercalation of the tetracene core, potentially enabling methylation of nearby guanine residues. Consistent with these structural insights, doxorubizen exhibited markedly higher cytotoxicity, mitochondrial depolarization, DNA intercalation, and cell-death induction compared with Dox. In fact, a Topo-II activity assay further confirmed its strong inhibitory effect. The mechanism of action of doxorubizen involves inhibition of DNA damage repair near double-strand breaks through guanine methylation, along with enhanced mitochondrial depolarisation and increased apoptosis. Moreover, in an acute leukaemia xenograft model, doxorubizen significantly reduced leukaemia burden relative to Dox while preserving body weight and liver function. Collectively, these findings highlight the promising therapeutic potential of doxorubizen for leukaemia treatment.

## 4. Conclusions

Methyl triazenes are among the earliest classes of alkylating agents introduced into clinical cancer therapy. In this review, we outline the evolution of these traditional prodrugs. While the first non-selective prodrugs lacked sufficient selectivity and ultimately did not gain clinical acceptance, they laid the groundwork for designing more advanced derivatives capable of tumour-targeted delivery. Notably, recently developed enzyme-activated methyl triazene derivatives provide a promising strategy, which has been summarised in Table 1. Moreover, the delivery of a second pharmacophore in addition to the methyl triazene clearly showed a potentiation in cytotoxicity, but the lack of tumour microenvironment activation of these triazene hybrids rendered them prone to unspecific release of the alkylating agent. Despite being at preclinical stages, these examples are setting up the research ground for future innovative activatable delivery systems of methytriazene hybrids to address the key limitations of the historical drugs DTIC and TMZ. On the other hand, the incorporation of TMZ into targeted nanosystems or the complexation of the tetrazine ring of TMZ with metals has been shown to be an alternative not only to improve the kinetic stability of TMZ but also to improve its solubility and delivery.

## Figures and Tables

**Table 1:** Comparison of different targeted strategies activated by the tumour microenvironment.

Activation	Promoeity	Linkage	Half-Life (*t*_1/2_)	Cytotoxicity
Tyrosinase	Tyramine based	Urea	PBS pH 7.4 = 0.25–15 days; 80% human plasma = 0.4–2 days; tyrosinase = 2.1–2.3 min	No cytotoxicity observed because no methyl triazene was released
	*N*-acyltyrosine based	Amide	PBS pH 7.4 = 42.2–72.2 h; 80% human plasma = 1.23–9.54 h; tyrosinase = 8.9–19.8 min	Showed limited cytotoxicity against MNT-1 cell line
	cysteaminylphenol based	Amide	PBS pH 7.4 = 3.6–5.8 days; 80% human plasma = 2.9–8.7 h; tyrosinase = 2.1–2.3 min	Comparable cytotoxicity to TMZ in MNT-1 and BF16F10 cancer cell lines
		Urea	PBS pH 7.4 > 10 days; 80% human plasma = 30.5–105 h; tyrosinase = 0.5–3.7 min	Higher cytotoxicity than TMZ in MNT-1 and BF16F10 cancer cell lines
Hypoxia	Nitroaryl based	Carbamate	Cell culture media > 2.5 days; nitroreductase = 10 to >60 min	Higher cytotoxicity than TMZ against U-87 cancer cell line but no significant difference between hypoxic and normoxic conditions

## Data Availability

No new data were created or analyzed in this study.
